# Immune response is a personal matter

**DOI:** 10.7554/eLife.00899

**Published:** 2013-07-16

**Authors:** Pedro G Ferreira, Emmanouil T Dermitzakis

**Affiliations:** 1 **Pedro G Ferreira** is at the Department of Genetic Medicine and Development, University of Geneva Medical School, Geneva, Switzerland, the Institute of Genetics and Genomics in Geneva, Geneva, Switzerland, and the Swiss Institute of Bioinformatics, Geneva, SwitzerlandPedro.DiasFerreira@unige.ch; 2 **Emmanouil T Dermitzakis** is an *eLife* reviewing editor, and is at the Department of Genetic Medicine and Development, University of Geneva Medical School, Geneva, Switzerland, the Institute of Genetics and Genomics in Geneva, Geneva, Switzerland, and the Swiss Institute of Bioinformatics, Geneva, Switzerlandemmanouil.dermitzakis@unige.ch

**Keywords:** Complex-trait genetics, Vaccines, Human genetics, Integrative biology, Systems biology, eQTL, Human

## Abstract

Changes in gene expression could be used to predict whether individuals will respond successfully to the influenza vaccine.

**Related research article** Franco LM, Bucasas KL, Wells JM, Niño D, Wang X, Zapata GE, Arden N, Renwick A, Yu P, Quarles JM, Bray MS, Couch RB, Belmont JW, Shaw CA. 2013. Integrative genomic analysis of the human immune response to influenza vaccination. *eLife* **2**:e00299. doi: 10.7554/eLife.00299

**Image** Many genes show altered expression in response to the influenza vaccine

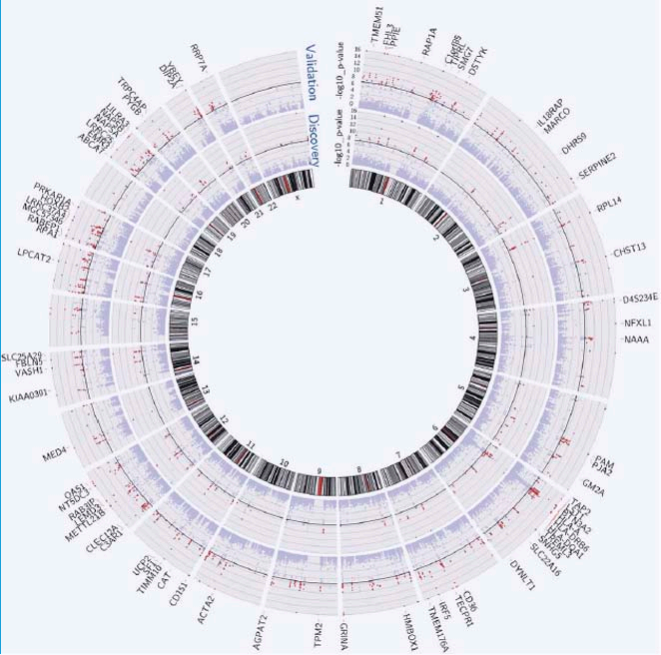

Seasonal influenza is estimated to kill up to half a million people every year according to the World Health Organization, and the death toll would be even higher were it not for the availability of a vaccine. However, individuals differ in their response to vaccination, with some producing relatively few antibodies and thus receiving limited protection against the influenza virus. Now, in *eLife*, John Belmont and Chad Shaw at Baylor College of Medicine and co-workers report new insights into the genetic and environmental factors that determine how an individual responds to the seasonal influenza vaccine (Franco et al., 2013). The effects of such factors can be probed by measuring any changes in gene expression (Morley et al., 2004; Stranger et al., 2007; Emilsson et al., 2008).

To examine the effects of influenza vaccination on gene expression, Belmont, Shaw and colleagues—including Luis Franco as first author—took blood samples from individuals before they were vaccinated against influenza, and at two time points afterwards. They sequenced the genomes of the individuals, and also measured the abundance of the corresponding mRNA transcripts at all three time points. In their first cohort of subjects, which consisted of 199 males, Franco et al. identified all the genes that showed altered transcription following vaccination, and where there was also evidence for genetic regulation of this effect by eQTLs (expression quantitative trait loci; these are genetic variants that regulate the expression of other genes; Figure 1). They were able to reproduce the majority of these associations in a second independent cohort of 128 females.

**Figure 1.**
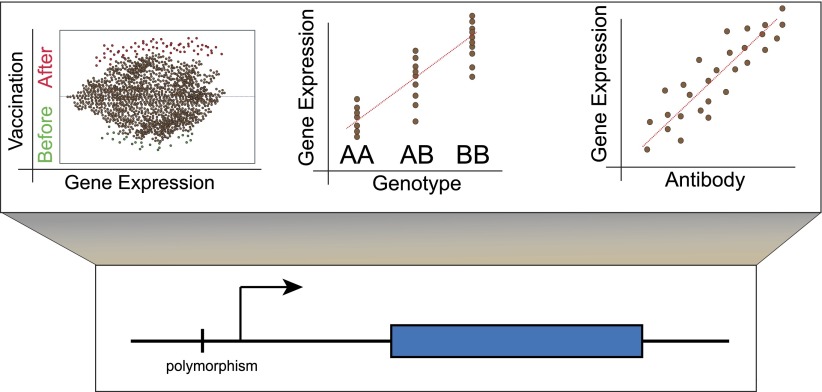
Lower panel: Genetic variants that regulate the expression of other genes are known as expression quantitative trait loci (eQTL) and they are typically found close to the transcription start site (arrow) of the gene that they regulate (blue rectangle). However, they also occur in exons and may thus affect the structure and stability of messenger RNA transcripts (Montgomery et al., 2010; Pickrell et al., 2010). Upper panel: Left, although the majority of genes do not show altered expression in response to vaccination, a minority do show different levels of expression before and afterwards. Centre, these changes in expression are influenced by the individual’s genotype at other loci. Right, for some genes, changes in expression correlate with the individual’s antibody response to vaccination. These genes could be used as biomarkers to predict whether an individual will respond successfully to a vaccine.

Using these data, Franco et al. then identified those genes for which the magnitude of the genotype effect had changed over time. Next they factored in antibody response to the vaccine, and determined which genetic loci showed changes in expression that correlated with the number of antibodies produced (Figure 1C). This narrowed down their list of genes to 20, each of which may have potential as a biomarker that could predict an individual’s response to the influenza vaccine.

Finally, Franco et al. applied specific statistical methods (Schadt et al., 2005) to their data to infer the relationship between genotype, gene expression levels and antibody response. The limited power of the analysis meant that they were unable to draw definitive conclusions about the causal nature of the relationship between these three components. Nevertheless, their results suggest a causal model in which an individual’s genotype influences their antibody response through changes in gene expression. The use of larger cohorts in future experiments will help to further elucidate these relationships and will avoid false positives resulting, for instance, from situations where the error in gene expression measurements is larger than that in antibody response measurements (Schadt et al., 2005).

A natural follow-up to this study would be to integrate data from other high-throughput sequence-based studies such as the ENCODE project (Dunham et al., 2012), which provide a wealth of information about various different elements across the genome. This will help us to understand the possible functional impact of eQTLs, especially those that fall in non-coding regulatory regions and correspond to transcription factor binding sites, chromatin marks or histone modification sites.

Application of high-throughput sequencing will provide further important insights into the transcriptome changes. For example, by quantifying the level of alternative splicing, it will be possible to detect transcriptional changes at the locus level and also at the transcript level. Additionally, it will become possible to detect sites where transcription occurs predominantly from one allele (Montgomery et al., 2010; Pickrell et al., 2010). We also anticipate a flurry of longitudinal studies in the near future: these might involve an external challenge, such as the vaccine in this study, or they might involve recording the changes in transcription that occur as a result of the natural environmental processes acting on an individual.

The genomic and transcriptomic biomarkers identified in this study are still preliminary and require further investigation, but they may make it possible to anticipate individual responses to vaccines, which would allow for a more targeted vaccination strategy. The study design demonstrates the power of longitudinal analysis to capture the complexity and the dynamics of human biological systems in the response to disease or environmental stimuli. It also represents yet another step toward personalized and precision medicine (Chen et al., 2012).
